# A Scoping Review Exploring Whether a Free “Offer” Devalues or Widens Sport and Physical Activity Participation Amongst Children and Young Adults Aged 0–25?

**DOI:** 10.3389/fspor.2022.897646

**Published:** 2022-05-11

**Authors:** E. J. Durden-Myers, L. Swaithes

**Affiliations:** ^1^The School of Education, Bath Spa University, Bath, United Kingdom; ^2^The School of Education and Humanities, The University of Gloucestershire, Cheltenham, United Kingdom; ^3^The School of Medicine, Keele University, Keele, United Kingdom

**Keywords:** sport, physical activity, widening participation, reducing inequalities, free offer

## Abstract

**Background:**

Socio-economic status continues to mediate physical activity engagement, despite a range of interventions aimed at reducing inequalities and widening sport and physical activity participation. As a result there has been increasing interest amongst policy makers, national governing bodies (NGB), county sports partnerships (CPS) and the sport and physical activity sector more broadly, in understanding how best to reduce inequalities and widen participation. The “price point” of offers and whether a “free offer” enables or devalues participation, has been a key area of interest. This scoping review aimed to explore this topic further by investigating whether “*a free “offer” devalues or widens sport and physical activity participation amongst children and young adults aged 0-25?”*.

**Methods:**

This scoping review searched three electronic bibliographic databases (MEDLINE, PsycINFO, SPORTDiscus) using a structured search strategy to identify articles published between 2017 and January 2022. Studies were included using the PICO criteria of; Population: children and young adults aged 0-25; Intervention: free “offer” relating to physical activity; Context: areas of deprivation in the UK; Outcome: engagement, involvement, participation in sport and physical activity.

**Results and Discussion:**

Five studies were eligible after screening 1301 titles and reviewing 14 full-text studies. Features reported included intervention design, outcomes, potential challenges and wider implications / future recommendations. Specifically, a narrative synthesis of the key themes of participation deprivation and cost effectiveness were outlined in more detail. A subsidized cost or free offer can improve participation generally and in attracting those from lower socio-economic backgrounds. However, the impact of such initiatives decrease with increasing deprivation highlighting that groups with the highest levels of deprivation have wider complexities affecting their participation. Competing priorities and potentially unrealistic expectations at stakeholders level was also identified.

**Conclusion:**

Despite the paucity of current research exploring the impact of a “free offer” in children and young adults, recommendations for future research, practice and policy included the need for longitudinal, more holistic and participant centric approaches. Further research is required to explore the impact of a “free offer” from an individual, societal and policy-level perspective, in widening and increasing participation in sport and physical activity.

## Background

Regular and sustained engagement in physical activity plays a major role in the promotion of health and wellbeing (Poitras et al., 2016; House of Lords, 2021). Regular engagement in physical activity has a variety of positive physical and psychological health and wellbeing benefits (Department of Health, 2011; Hills et al., 2015). One of the key public health goals in the UK is to increase engagement in regular physical activity within the population. As a result, there has been a plethora of policies within the UK (Department of Health, 2003, 2004, 2005, 2009, 2010a,b, 2011) and resultant interventions that have sought to improve participation in sport and physical activity, results however, have often been disappointing (Rabiee et al., 2015; House of Lords, 2021). Throughout this paper we use the term sport and physical activity participation to include “*experiences in physically demanding movement, sport, game, or recreational play that results in energy expenditure and perceptions of communal involvement*” (Ross et al., 2016; P.8).

Physical activity levels among members of minority ethnic groups and lower socio-economic groups continue to be particularly low (Department of Health, 2011). Individuals from lower socio-economic groups are also five times more likely to engage in unhealthy behaviors such as smoking, excessive alcohol use, poor diet and low levels of physical activity (Rabiee et al., 2015). This picture also extends to childhood participation in sport and physical activity (Sport England, 2021). Current World Health Organization (World Health Organisation, 2010) and UK physical activity guidelines (Department of Health, 2019) recommend that school-aged children should spend at least 60 min per day in moderate-to-vigorous physical activity. However, only 44.6% of UK children met these guidelines, with children from the least affluent families lower again at 39% (Sport England, 2021). As a result socio-economic status continues to mediate physical activity engagement (Department of Health, 2011; Rabiee et al., 2015; House of Lords, 2021;Sport England, 2021).

As a result there has been a targeted effort to widen participation in sport and physical activity and reduce inequalities, especially for those from areas of deprivation and increase participation for lower socio-economic groups, in the UK. For example, the 4 million pounds “Be Active Wales Fund” from Sport Wales (2020a) aimed to protect and progress community sport. Specifically, the progress fund aims to progress sport and activity to the next step and support long-term sustainability. This grant is intended to help clubs and community organizations: Tackle inequality, create long-term solutions to be more sustainable and take innovative approaches (Sport Wales, 2020a). Sport England (2020) has also devoted funding to specifically tackle inequalities in sport and physical activity participation through the Tackling Inequalities Fund (TIF). TIF was set up in April 2020 to help the sport and physical activity sector through the COVID-19 pandemic. TIF was created with £20 million of National Lottery funding to try and help reduce the negative impact on activity levels in these under-represented groups, with a specific focus on: Lower socio-economic groups, Culturally diverse communities, Disabled people, people with long term health conditions. The fund has now been extended and renamed the “together fund” and will run until March 2023 (Sport England, 2020). There have been a number of recent successful interventions that incorporated “free” offers within the adult population such as “Gym for Free” in Birmingham (Rabiee et al., 2015) and “Leeds Let's Get Active” (Candio et al., 2020), yet little is known about the effectiveness of such interventions for young people aged 0–25.

The Welsh “Sport and Active Lifestyles Survey” (2019–2020) (Sport Wales, 2020b) also reported that in 2019–20, just prior to Coronavirus, 32% of adults (16+) participated in a sporting activity three times a week or more(808,000 people). 7% participated approximately twice a week (186,000 people), 11% participated approximately once a week (268,000 people) and 50% participated less than once a week (1,245,000 people). Moreover, 41% of adults had not participated in any sporting activity (1,040,000 people) in the previous 4 weeks. This data highlights how there is an opportunity to increase regular sport participation to over 1 million adults in Wales who are currently not engaging regularly in sport participation. In 2021, Sport Wales established a “foundation and participation” group, that consisted of representatives from national NGBs, SPs and wider stakeholders, with the aim of increasing and widening sport and physical activity participation. The notion of a free offer was a frequent topic of interest during meetings with mixed views on whether it would enable or devalue sport and physical activity participation. This scoping review was requested by the Sport Wales “foundation and participation” group to find out whether “*a free “offer” devalues or widens sport and physical activity participation amongst children and young adults aged 0*–*25?”*. Scoping reviews allow for a broader conceptual scope of the literature (Arksey and O'Malley, 2005) and can stimulate new research questions that can further advance research in this area (Peterson et al., 2017). A specific focus on children and young people was requested to draw out recommendations for provision early in life supporting children and young adults in accessing opportunities to support their future adult behaviors. The findings of this review were presented back to the group to inform their future work.

## Methods

A scoping review was conducted guided by Arksey and O'Malley's five-step methodological framework: (1) identifying the research question, (2) identifying relevant studies, (3) study selection, (4) charting the data, and (5) collating, summarizing, and reporting the results (Arksey and O'Malley, 2005). This manuscript has also been prepared adhering to the PRISMA extension for scoping reviews (PRISMA-ScR Appendix A, Tricco et al., 2018).

### Research Question

The research was guided by the following research question “*Does a free ‘offer' devalue or widen sport and physical activity participation amongst children and young adults aged 0–25?”*. The findings were used to inform future interventions amongst county sport partnerships and National Governing Bodies in Wales.

### Identification of Relevant Studies: Search Strategy

Prior to the database searches, a preliminary literature search was undertaken by searching MEDLINE using keywords, to gauge the volume and type of literature available, inform a more comprehensive search strategy and list potential eligibility criteria. A stakeholder engagement meeting with Sport Wales was conducted to expand and refine the potential search terms (Appendix B). Previous scoping and literature reviews were also screened to check for database selection, which were subsequently used to inform the selection of databases to search.

A search strategy was formulated using the PICO (population, intervention, context, outcome) framework. The *population* of interest was young people aged 0–24. Relevant *interventions* include any physical activity intervention that was subsidized, funded or free. Areas of deprivation in the UK represented the *context* of interest. *Outcomes* included (but were not limited to) participation, involvement, and experiences. Three electronic bibliographic databases (MEDLINE, PsycINFO, SPORTDiscus) were searched (see Supplementary Material) to identify articles published between 2017 and 31st of January 2022. Reference lists of included articles were also checked. A 5 year period was requested by the foundation and participation group to capture “contemporary” research in the pre and post COVID-19 landscape.

### Study Selection

Table 1 describes the inclusion and exclusion criteria for this review. One author conducted title and abstract screening (LS). In the instance of uncertainty for study eligibility, the full text article was obtained and added to the full text screening phase for further clarification. Each of the full text articles were reviewed by two researchers (EDM, LS).

**Table 1:** Criteria for including studies in the review.

	**Inclusions**
Population	To include young people aged 0–24
Intervention	Focus on physical activity interventions which are free, subsidized or funded
Context	Areas of deprivation in the UK
Outcome	May include participation, engagement, experiences, factors relating to cost, or impact
	**Exclusions**
Population	No participants aged 0–24 (e.g., studies focusing on physical activity in retired populations)
Intervention	Studies that include other interventions (e.g. smoking cessation, nutrition)
Context	Non-uk; affluent areas
Outcome	No mention of any of the key outcomes listed in the inclusion criteria

### Charting the Data

Key information obtained from each study included the author, year of publication, nature of the intervention, population and study setting, methods, key findings and recommendations, which were captured in a table.

### Collating, Summarizing and Reporting the Results

A narrative synthesis was conducted of the included papers. “Narrative synthesis” refers to an approach synthesizing findings from multiple studies that relies primarily on the use of words and text to summarize and explain the findings of the synthesis. Whilst narrative synthesis can involve the manipulation of statistical data, the defining characteristic is that it adopts a textual approach to the process of synthesis to “tell the story” of the findings from the included studies. As used here “narrative synthesis” refers to a process of synthesis that can be used in scoping reviews focusing on a wide range of questions, not only those relating to the effectiveness of a particular intervention (Popay et al., 2006). Several methods were utilized to enhance the review's validity, including using multiple researchers in the development of search terms, peer debriefing, strict inclusion and exclusion criteria in performing searches, and in the selection and analysis of papers.

## Results

**Figure 1:**
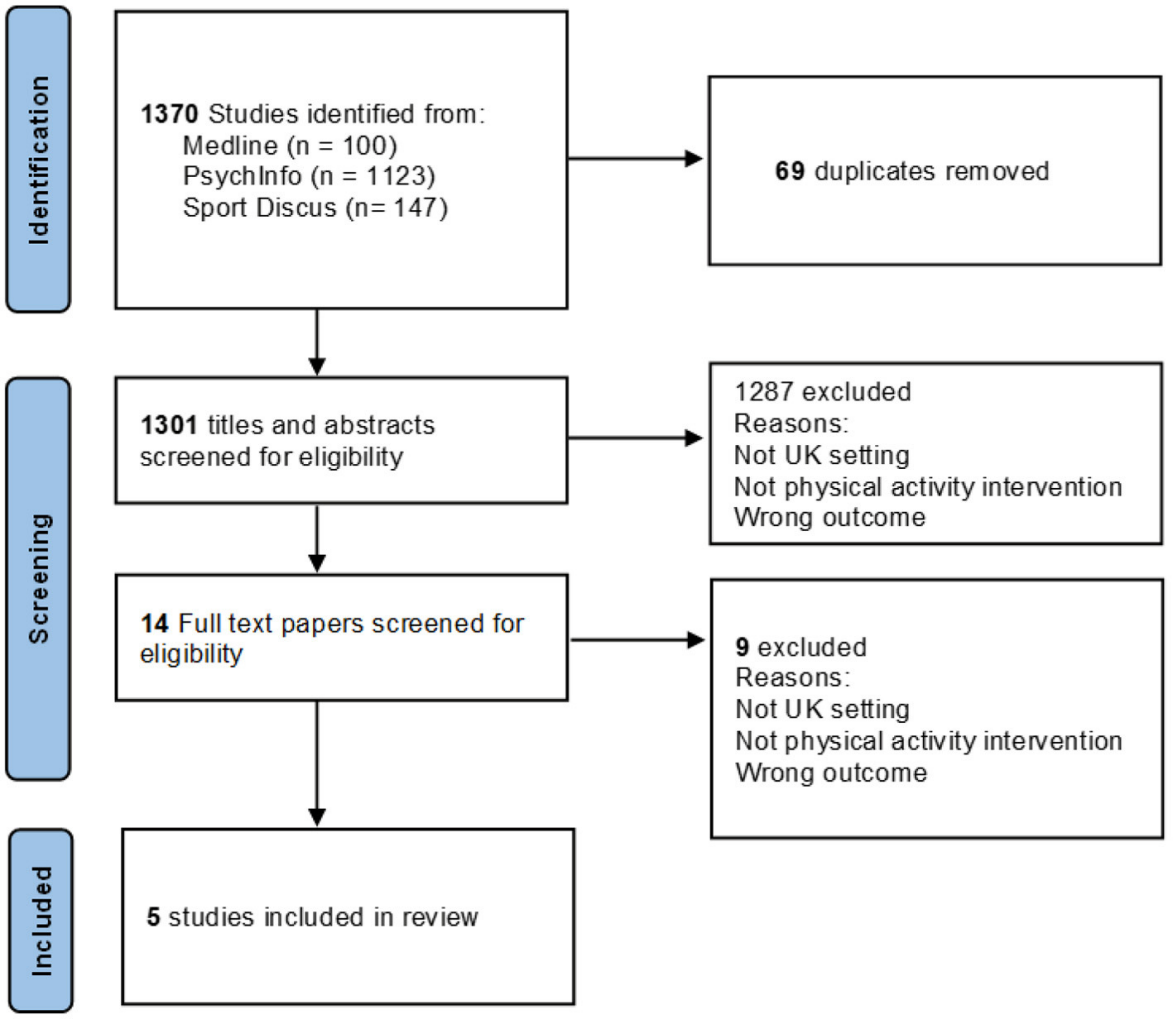
Flowchart documenting the study selection process for the review.

The searches identified 1,370 titles, leaving 1,301 after deduplication. The screening of titles and abstracts reduced the number of papers from 1,301 to 14. Of the remaining 14 full text papers, nine were excluded for reasons including non-UK context, no physical activity intervention and, wrong outcome of interest, leaving five papers eligible for the review. Figure 1 sets out the review process in a flowchart. An overview of the five studies included in the review and a description of the intervention and the key findings is presented in Table 2.

**Table 2:** Study characteristics and data charting.

**Study**	**Intervention**	**Population/** **setting**	**Study aim**	**Methods**	**Key findings**	**Recommendations**
Higgerson et al. (2018a)	Free swimming access during school holidays	Children aged 5–15 Highly disadvantaged local authority in Northwest England (Blackpool)	To investigate the extent to which providing children with free swimming access during school holidays increased participation and whether this effect differed according to socioeconomic deprivation of the neighborhoods.	Comparative regression discontinuity (intervention local authority V control local authority). Estimated differential effect of intervention across five groups, defined by quintiles of area deprivation.	Free swimming during the summer holidays was associated with an additional 6% of children swimming and an additional 33 swims per 100 children per year. The effects were greatest within areas with immediate levels of deprivation (quintiles 3 and 4) within the deprived local authority.	Providing free facilities for children in disadvantaged areas is likely to increase swimming participation and may help reduce inequalities in physical activity
Higgerson et al. (2018b)	Re:fresh scheme – began in 2008 providing free access to activities across nine leisure centers (swimming pools and gyms) at most times of day along with community outreach activities. The intervention included outreach and marketing activities. In 2016, a flat fee of £1 was introduced.	Initially the intervention was available to people ages over 50 years old. It was then extended to people aged 16–24, and again to those aged 25–49. Deprived local authority area in Northwest England	To investigate the impact of an intervention providing universal free access to leisure facilities alongside outreach and marketing activities	Quasi-experimental methods - Interrupted time series and difference-in-differences analyses of local administrative data and a large national survey	The intervention was associated with: A 64% increase in attendances in swimming and gym sessions An additional 3.9% of the population participating in at least 30 min of moderate intensity gym or swim sessions during the previous 4 weeks An additional 1.9% of the population participating in any sport or active recreation of at least moderate intensity for at least 30 min on at least 12 days of the last 4 weeks The effect on gym and swim activity and participation in physical activity was significantly greater for the more disadvantaged socio-economic group	The study suggests that removing user charges from leisure facilities can increase overall population levels of physical activity while reducing levels of inequality, when implemented in combination with outreach activities (delivered by Health Trainers and a healthy Community Partnership who offered 1:1 and group sessions to provide taster sessions and support behavior change through goal setting and motivational interviewing) and marketing and promotional activities.
Candio et al. (2020)	Leeds Lets get Active (LLGA) – provision of universal access to free off-peak City Council leisure center-based exercise sessions to all city residents. Exercise sessions included the use of the free weights areas, swimming pool access and fitness classes. The intervention ran for 39 months from Oct 2013 to end Dec 2016.	551,874 residents aged 16 and over The North of England located in the most deprived areas of the city of Leeds Data was comprised of responses from 16–40yr - 61.5% 41–64yr - 31.5% Over 64–7%	To assess the cost-effectiveness of a proportionate universal programme to reduce physical inactivity	Continuous-time Markov chain model developed to assess the cost implications and QALY gains associated with increases in physical activity levels across the adult population. Baseline model data obtained from previous economic models, population-based surveys and other literature	A non-negligible level of uncertainty, regarding the effectiveness and therefore, cost-effectiveness of a universal offer of free leisure center-based exercise that targets hard to reach groups A proportionate universal offer of free off-peak exercise in public leisure centers can provide good value for money	Local governments should evaluate the possibility of providing universal access to off-peak exercise sessions in public leisure centers
Candio et al. (2022)	As above	As above	To evaluate the LLGA programme in terms of reach and efficacy and how these varied across population groups	Descriptive statistics used to summarize program data and participants Time to event, count and logistic regression models examined how different population subgroups engaged with the program (number of entries, weekly participation rates and drop-off patterns)	Of the 51,874 adults who registered to the program only 1.6% attended the free sessions on a weekly basis. Higher participation rates were estimated for groups of males, retired and non-inactive participants. A neighborhood-level deprivation status was found to have no marginal effect on the level and frequency of participation, but to be negatively associated with participation drop-off	Providing everyone with free-of-charge organized exercise opportunities in public leisure centers located in deprived areas can attract large volumes of residents, but may not sufficiently encourage adults, especially inactive residents and those living in disadvantaged neighborhoods to take up regular exercise
Ward et al. (2017)	Current leisure center offer (some areas included a free offer for children)	83 participants aged 18 and above Four local authorities in the Northwest of England, in areas of significant socio-economic deprivation	To investigate public perceptions of entrance charges and how the charges influenced participation by adults living in lower income neighborhoods	Qualitative study – focus groups and interviews	Cost was a key factor which influenced physical activity participation in low-income neighborhoods Pre-paid options (direct debit memberships) encouraged participations Entrance charges incurred each time an individual participated had a negative impact of frequency but were a convenient way of paying for occasional use or for people who were unable to afford a pre-paid option Free access helped people who could not afford pre-paid membership to exercise regularly as well as incentivizing non-users to try activities	Public organizations that commission or deliver physical activity interventions and services should consider options that enable people to afford more easily to participate in a wider range of activities. This could include cheaper PAYG options for those who cannot commit to pre-paid membership, free sessions at a range of times and affordable provision at peak times for those on low-incomes.

### Results Overview

Whilst each study explored interventions or current provision that included a “free offer” (Higgerson et al., 2018a,b; Candio et al., 2020, 2022) or perceptions of free and subsided offers (Ward et al., 2017) for swimming, gym, and/or fitness classes they all had different aims and outcomes, including sport and physical activity participation, cost-effectiveness, reach and efficacy. The duration of each intervention varied from a few weeks (during school holidays (Higgerson et al., 2018a), 39 months (Candio et al., 2020, 2022), to several years (Higgerson et al., 2018b).

Higgerson et al. (2018a) investigated the extent to which providing children with free swimming access during school holidays increased sport and physical activity participation and whether this effect differed according to socioeconomic deprivation of the neighborhoods in which children lived. Higgerson et al. (2018b) investigated the impact of an intervention providing universal free access to leisure facilities alongside outreach and marketing activities. Candio et al. (2020, 2022) analyzed the Leeds Lets get Active (LLGA) provision of universal access to free off-peak City Council leisure center-based exercise sessions to all city residents. Candio et al. (2020) focussed on the assessment of cost-effectiveness of the LLGA programme to reduce physical inactivity while Candio et al. (2022) evaluated the LLGA programme in terms of reach and efficacy and how these varied across population groups. Finally, Ward et al. (2017) investigated public perceptions of entrance charges and how the charges influenced sport and physical activity participation by adults living in lower income neighborhoods. Two main themes emerged that warrant further discussion pertaining to 1) sport and physical activity participation and deprivation and 2) cost effectiveness.

### Participation and Deprivation

Increased participation was described as an outcome in all of the five studies. The study by Higgerson et al. (2018a) was the only paper identified that reported on increasing participation specifically for children and adolescents from disadvantaged backgrounds. The study investigated the extent to which providing children (5–18) with free swimming access during school holidays increased sport and physical activity participation and whether this effect differed according to socioeconomic deprivation of the neighborhoods in which the children lived. The intervention took place within a disadvantaged local authority in Northwest England (Blackpool). The results found that offering free swimming during the summer holidays was associated with an additional 6% of children swimming and an additional 33 swims per 100 children per year. The effects were greatest within areas with immediate levels of deprivation within the deprived local authority, and were greatest amongst the were greatest amongst children aged 10–14 years olds.

The estimates made by Higgerson et al. (2018a) were greater than those reported in the evaluation of the national free swimming programme (an additional 1% of children swimming as a result of the free offer), which is significant because the national programme evaluation only looked at average effects across the country.

The search strategy identified two further papers (Higgerson et al., 2018b; Candio et al., 2022) that reported on participant engagement in free to access offers. These studies were typically aimed to engage the adult population and then altered to include younger participants. Higgerson et al. (2018b) refresh scheme began in 2008 providing free access to activities across nine leisure centers (swimming pools and gyms) at most times of day along with community outreach activities. The intervention was associated with a 64% increase in attendances in swimming and gym sessions. The effect on gym and swim activity and overall levels of participation in sport and physical activity was significantly greater for the more disadvantaged socio-economic group. The study suggests that removing user charges from leisure facilities can increase overall population levels of physical activity while reducing levels of inequality, when implemented in combination with outreach activities (delivered by Health Trainers and a healthy Community Partnership who offered 1:1 and group sessions to provide taster sessions and support behavior change through goal setting and motivational interviewing) and marketing and promotional activities.

Candio et al. (2022) reported that of the 51,874 adults who registered to the programme only 1.6% attended the free sessions on a weekly basis. Higher participation in sport and physical activity rates were estimated for groups of males, retired and non-inactive participants. A neighborhood-level deprivation status was found to have no marginal effect on the level and frequency of participation, but to be negatively associated with participation drop-off. Providing everyone with free-of-charge organized exercise opportunities in public leisure centers located in deprived areas can attract large volumes of residents, but may not sufficiently encourage adults, especially inactive residents and those living in disadvantaged neighborhoods to take up regular exercise.

### Cost Effectiveness

Four of the five studies included free to access offers with Ward et al. (2017) exploring both free and subsidized access. Only one study (Candio et al., 2020) evaluated cost effectiveness (in the longer term) with the others reporting on shorter term sport and physical activity participation rates. Only one study (Ward et al., 2017) reported the perceptions of cost-effectiveness from the perspective of users. Findings in relation to cost-effectiveness from Ward et al. (2017) and Candio et al. (2020) are expanded upon below.

Results from Candio et al. (2020) indicate that LLGA is highly likely to be cost-effective under base-case assumptions. The net benefits of implementing LLGA increase as a longer time horizon is considered. Scenario analyses also show that identification of the optimal strategy is highly dependent on variations to key structural elements regarding the sustainability of the intervention effect over time and assumed mechanisms of survey non-response. Specifically, in relation to deprivation Candio et al. (2022) found that providing everyone with free-of-charge organized exercise opportunities in public leisure centers located in deprived areas can attract large volumes of residents, but may not sufficiently encourage adults, especially inactive residents and those living in disadvantaged neighborhoods, to take up regular exercise. They suggest that inactivity within areas of deprivation is more multidimensional than just creating free or subsidized opportunities.

Only one study (Ward et al., 2017) reported the perceptions of those whom the “free offer” was intended for, however, young people under 18 years were excluded from this study as it was anticipated that in most cases, parents/guardians would be responsible for paying entrance charges. The qualitative study by Ward et al. (2017) identified that the connection between cost and participation was multifaceted and influenced by factors such as motivation, value and affordability. Whilst the study did not solely explore a specific “free offer” intervention, focus groups and interviews were conducted with 83 public participants to identify perceptions of both free and subsidized offers and how these influenced participation in four local authorities.

With regards to subsidized access to leisure, findings suggest that concessionary rates were inadequate for those on low incomes and that price was a barrier to sport and physical activity participation which, in some cases, posed “*a choice between exercising and eating”*. In addition, subsidized access was reported to be largely available “off-peak”. A consequence of this was that participants with studying, work, or childcare commitments did not attend at all.

Findings relating to the value of free leisure access were mixed. Free access helped people who could not afford pre-paid membership to exercise regularly as well as incentivizing non-users to try activities. Some participants described how removing the price barrier through the provision of free sessions encouraged attendance for inactive people, and in some instances, resulted in more regular attendance. However, in contrast, some participants suggested that a “free offer” might not be valued and would therefore not result in regular sport and physical activity participation (“*I don't have to bother because it doesn't cost me anything”*). Whilst free sessions were valued by those who utilized them, the financial implications to the service provider were acknowledged by some participants.

Across several focus groups participants shared a view about the role of free swimming (which was available for all children in one area and during school holidays in others) to encourage more children to exercise. Free swimming was seen to be particularly important in getting parents to exercise with their children. However, the withdrawal of the free swimming offer in another locality was seen as a significant loss as people felt that a lot of families would not be able to afford to pay for swimming, particularly if they had more than one child.

## Discussion

This scoping review has explored an important area in physical activity research to identify the impact of a “free offer” in UK research. Despite a paucity of UK studies, five studies were included in the narrative synthesis. Findings indicate that free and subsidized offers can be effective in increasing sport and physical activity participation although this depends on a number of wider factors including type of physical activity/sport, target population, degree of deprivation, target age, family and individual employment status (16–25), additional protected characteristics such as disability, and environmental factors such as changing facilities and provision.

### Widening and Increasing Participation for Children and Young People

The conclusion by Higgerson et al. (2018a) conclude and recommend that providing free facilities and access to opportunities for children from disadvantaged areas, is likely to increase swimming participation and may help to reduce inequalities in sport and physical activity participation is also supported by other studies. Higgerson et al. (2018b) also indicate that removing user charges from leisure facilities in combination with outreach and marketing activities could potentially increase overall levels of physical activity while reducing inequalities. Refresh may have achieved lower inequalities in sport and physical activity participation due its universal nature, and availability of sessions during 90% of opening hours, therefore including people on low incomes who work full-time, who might be excluded from other more targeted schemes (such as the provision of cheaper facilities for those in receipt of state benefits) or only during off-peak hours.

As Higgerson et al. (2018a) outline, it is plausible that the effect of free swimming is greater in more deprived areas and the visibility of this impact can be lost when comparing the national average effect of a free swimming offer across the whole country. However, Higgerson et al. (2018a) also report that the effect size decreased as the severity of deprivation increased. This may be as a result of the population also experiencing multiple forms of deprivation for example. Poverty, disability, poor housing, unemployment. Given the multiple issues surrounding some families in these very deprived areas it is perhaps not surprising that the free swimming offer had a smaller effect on participation. Therefore, those in most need, may in fact require more support than just the removal of cost to access. For example, further funding may also be required to provide swimming lessons, equipment and swim suits for those who experience significant levels of deprivation as cost to access alone will not solve these wider cost implications.

Candio et al. (2020) however is more cautious highlighting the intrinsic complexity of PA behavior and the even greater complexity around impact capture, analysis and evaluation. They call for analysis to be embedded within intervention and programme design to better integrate the evaluation of projects and programmes in the pursuit of more valuable research outputs, as well as to support the building of local research and implementation capacity.

Candio et al. (2022) also presents a more complex conclusion which discusses the challenges associated with encouraging long term adoption of regular exercise. They argue that providing everyone with free-of-charge organized exercise opportunities can attract large volumes of adult residents but are likely to encourage only a selective minority to take up regular exercise. While removing user charges can be a tempting strategy, it alone is not sufficient to promote sustained sport and physical activity participation at a population level. Universal policies do not achieve the desired outcome of supporting already inactive adults, hence alternative approaches should be considered. Unstructured involvements of research professionals limit the ability to adequately design, conduct and evaluate these interventions and their impact on health outcomes and inequalities. With increasing pressure on local government budgets, established collaborations with academic institutions would help support an efficient allocation of public health resources by adequately informing policy decision-making (Candio et al., 2022). This more complex view is also shared by Ward et al. (2017) who states that cost is one of the factors which influences levels of PA in low income neighborhoods but also highlights the importance of the range of entrance charges that are available when considering the impact of price on the use of LA leisure centers. The research also found that price can be important in encouraging sustained levels of PA, including for people who were previously physically inactive.

Additionally there were two noteworthy articles that were excluded during the screening process due to the research not meeting the inclusion criteria of being an intervention within the 0–25 demographic. These research articles instead, explored the stakeholder perceptions namely at the local government (Candio et al., 2021) and local authority level (Halliday et al., 2018) of providing cost effective incentives to promote physical activity engagement.

Candio et al. (2021) took a local government perspective for economic evaluation and articulated how this approach may affect economic conclusions. This research highlights the wider metrics, voices and actors guiding, informing and evaluating success in this arena. An awareness of the information and the metrics valued by decision makers and potentially budget holders is key, in understanding the wider landscape of promoting physical activity. In relation to our research question “Does a free “offer” devalue or widen sport and physical activity participation amongst children and young adults aged 0-25?” It could be extended to include “… and at what cost” in light of Candio et al. (2021) research. There may also be some important awareness advocacy work to be conducted in this space as the true cost may be far more than many may estimate. Candio et al. (2021) in their research found from a local government perspective, attracting a local resident to the programme (i.e. registration) was estimated at a cost of £2.77 per person/year. The average cost of achieving the goal of a resident attending at least one programme session was £6.11, whereas 3.8 times as much would be necessary for an inactive adult to engage. The average cost of moving an inactive resident to an active state was substantially higher, at £1,406.78 per year. Around 4.7 times as much would be necessary for achieving these goals for adults living in the most deprived areas. This highlights how expectations, especially those relating to cost of interventions when working with participants from areas of deprivation should be significantly different from general population interventions, therefore perhaps the amendment of “… and at what relative cost” should be included within this research area.

Halliday et al. (2018) explored the perspectives of 33 leisure and public health professionals from 7 local authorities in Northwest England. They investigated the different approaches to pricing (facility charges) and the rationales that influenced the decision making. Halliday et al. (2018) offer some insights into the challenges of joint working for public health teams embedded within complex socio-political and economic environments. The results found that welfare orientated (e.g. affordability) and commercial drivers (e.g. income generation) featured most prominently across areas. Pricing policies placed less direct focus on public health goals, although tackling inactivity was articulated as part of leisure's role more generally. Local targeting of free/concessionary offers was also defined and implemented differently. Decision makers described navigating competing pressures of providing services for the public “good” yet remaining financially viable.

Halliday et al. (2018) offer evidence of how pricing decisions are made and the approaches adopted in practice as well highlighting the conflicting priorities for decision makers especially within an austerity context. This is significant because if need or pressure for profitability and income generation outweighs public good then subsidized or free offers are not likely to be favorable at the decision making level. Especially as Candio et al. (2021) highlights reaching the deprived and inactive can be 4.7 times more costly. Therefore, this research encourages us to ask to what extent is public good, including widening participation in sport and physical activity and reducing inequalities valued in comparison to other factors or competing priorities such as income generation.

Both Higgerson et al. (2018b) and Candio et al. (2022) expressed estimates and expectations that were much higher than the actual impact on sport and physical activity participation in reality. This further supports that there might be an over-expectation of the impact free offers may have from key stakeholders in widening participation. While both Higgerson et al. (2018b) and Candio et al. (2022) did report improvements in sport and physical activity participation especially within areas of deprivation both argued that widening participation sustainably and for the inactive may not be as simplistic as creating a free to access offer, although this is a significant barrier for some. The over estimations and preconceived notions of effectiveness of increasing sport and physical activity participation with free offers especially for those who are inactive and from areas of deprivation (Higgerson et al., 2018b; Candio et al., 2022) demonstrates a naivety or perhaps visible privilege at policy and decision making level, with an assumption of needs and wants of the participants from the populations with complex societal needs and barriers. This coupled with the competing priorities and lessening resources makes for a difficult situation. Higgerson et al. (2018b) highlight that local authorities in the UK are facing severe cuts to their budgets while being granted greater responsibilities for promoting public health and reducing health inequalities. Local authorities are therefore having to make difficult decisions about the targeting of resources to those interventions that are likely to have the most impact. Public organizations that commission or deliver physical activity interventions and services should consider options that enable people to afford more easily to participate in a wider range of activities. This could include cheaper PAYG options for those who cannot commit to pre-paid membership, free sessions at a range of times and affordable provision at peak times for those on low-incomes (Ward et al., 2017). For others including women from ethic minority backgrounds and people with a physical disability, knowledge about the activities offered and the physical environment (including accessibility in the pool areas, privacy in the changing rooms and women only sessions) were reported to have as great a bearing on sport and physical activity participation decisions as the cost of attending (Ward et al., 2017).

### Strengths and Limitations

This scoping review used rigorous methods including the use of published guidance on the conduct of scoping reviews (Arksey and O'Malley, 2005) and the PRISMA extension for scoping reviews (PRISMA-Scr, Tricco et al., 2018). Two reviewers were involved in the development of search terms (along with key stakeholders) and eligibility criteria, conducting the searches and in the selection and analysis of papers. In line with guidance for conducting scoping reviews, quality appraisal of the included studies was not conducted, however it is recognized that this would have provided the reader with additional information relating to the trustworthiness of the results. For example, the Candio et al. (2022) paper had a significant number of participants (51,874 residents aged 16 and over with participants aged between 16–40 representing 61.5% of the results) but it failed to report on how many participants could be included within the 16–25 age range specifically. Searching databases for published research is another potential limitation of this scoping review as searching gray literature may have yielded valuable reports in the topic area. Another limitation included the date range of the inclusion criteria (between 2017 and 2022) as although these papers were published this date range many of the studies were conducted prior to this period. A wider or more flexible date range and may have revealed further results.

### Recommendations and Implications for Policy, Practice and Research

This scoping review has identified gaps in current literature and several implications for policy, practice and further research. We recommend that more research specifically focussed on children and young adults aged 0–25 in the UK is required. This should include public participant involvement whereby key questions are identified and informed by key stakeholders to ensure research is relevant to both policy makers, organizations and the end user. Universal approaches revealed both positive (Higgerson et al., 2018b) and negative (Candio et al., 2022) findings therefore more research is required to ascertain whether targeted or universal offers are more effective at widening sport and physical activity participation. Ward et al. (2017) support this view in their recommendation for further qualitative work located in socioeconomically diverse neighborhoods investigating attitudes to leisure facilities and PA more generally among all groups who do not meet recommended activity levels. They go further to state that public organizations that commission or deliver PA interventions and services should consider options that enable people to more easily afford to participate in a wider range of activities. This could include cheaper PAYG options for those who cannot commit to pre-paid membership, free sessions on offer at a range of times and affordable provision in peak-times for those on low incomes or who work “non-regular” hours. However, leisure services and interventions may also risk increasing inequalities if barriers to accessibility and acceptability for different groups are also not adequately addressed alongside cost.

There is also little longitudinal research looking at sustained sport and physical activity participation over longer periods of time or interventions with inbuilt follow up protocols. More holistic approaches are also advocated including mixed methods studies that encompasses both economic evaluation, key stakeholder experiences, sport and physical activity participation and address both the localized and national picture to understand the provision available across different regions/countries in the UK as currently we don't have an understanding of what features of interventions work in certain demographics/localities.

The studies identified also didn't report on a retention or maintenance strategy, once participants are recruited and are participating how is this engagement retained? We recommend that wider research be drawn upon such as Norris et al. (2018) and their use of the RE-AIM framework criteria which aims to more holistically consider provision by considering Reach, Effectiveness, Adoption, Implementation and Maintenance.

In view of the changes in physical activity provision that have insured during the COVID-19 pandemic, further research is also needed to explore the landscape of a “virtual free-offer” and to identify the impact of this on sport and physical activity participation and end user experience. Drawing upon research such as Norris et al. (2018) delivering online and virtual opportunities may also widen sport and physical activity participation but not in the traditional form (footfall in sport and leisure settings). This is supported by Ward et al. (2017) who recommend further research with those who exercise at home or at community/private facilities and classes to understand why they chose these options rather than LA facilities. It is important to understand if a virtual approach to physical activity provision widens access or instead promotes inequality by excluding those without digital skills or access. A more comprehensive understanding of where, what and how children and young people may be active and how these range of opportunities can be designed with inclusivity and widening participation in sport and physical activity as a central focus is recommended. We recommend that a subsequent “systematic review” be undertaken to extend these initial findings from this “rapid scoping review” and may address many of these limitations by searching wider databases, gray literature and include a quality appraisal process. Widening the inclusion criteria to beyond the UK may also return insights from international research and wider contexts, that may also be used to compare whether this scoping review results are consistent or different from other geographical contexts.

Finally, we recommend that caution be exercised when balancing competing priorities at a local decision making level. This is supported by Higgerson et al. (2018b) who argue that with the increasing cuts to local government budgets in the UK, many councils are considering whether to reduce the public subsidy of leisure facilities and discontinue the free leisure schemes that currently exist. There is also the potential for other funding such as local government public health grants or health service funds to be invested in subsidizing leisure facilities to promote public health. Higgerson et al. (2018b) provides evidence that expanding free leisure schemes is likely to increase physical activity and reduce inequalities, while discontinuing these schemes may have the opposite effect. As such, elevating the value and importance of free offers with key stakeholders while not over estimating their impact is essential in ensuring cost does not become the significant barrier for widening participation in sport and physical activity.

## Conclusion

This scoping review has highlighted that while there are free and subsidized offers that exist within the adult and young adult population, little research has been conducted within childhood. With childhood behaviors often tracking into adulthood, increasing and widening participation early is a significant opportunity to change the landscape of participation in sport and physical activity more broadly. Socio-economic status and the cost of opportunities continue to be mediating factors (among others) of participation in sport and physical activity. The research reveals mixed perspectives with regards to whether free offers can significantly improve participation in sport and physical activity. Further research is required to explore the impact of a “free offer” from an individual, societal and policy-level perspective, in widening and increasing participation in sport and physical activity.

## Author Contributions

ED-M was the lead author on this paper with research support being provided by LS to conduct peer checking and database searches/cross referencing analysis. Both authors contributed to the article and approved the submitted version.

## Funding

This scoping review has been funded by Sport Wales, with the publication fee funded by PE Scholar (www.pescholar.com).

## Conflict of Interest

The authors declare that the research was conducted in the absence of any commercial or financial relationships that could be construed as a potential conflict of interest.

## Publisher's Note

All claims expressed in this article are solely those of the authors and do not necessarily represent those of their affiliated organizations, or those of the publisher, the editors and the reviewers. Any product that may be evaluated in this article, or claim that may be made by its manufacturer, is not guaranteed or endorsed by the publisher.
